# Comparison of Sinus Deposition from an Aqueous Nasal Spray and Pressurised MDI in a Post-Endoscopic Sinus Surgery Nasal Replica

**DOI:** 10.1007/s11095-021-03129-2

**Published:** 2022-02-08

**Authors:** Joey Siu, James van Strien, Rosa Campbell, Paul Roberts, Malcolm Drummond Tingle, Kiao Inthavong, Richard George Douglas

**Affiliations:** 1 Department of Surgery, The University of Auckland, Private Bag 92019, Auckland, 1142 New Zealand; 2 Mechanical & Automotive Engineering, School of Engineering, RMIT University, Bundoora, VIC Australia; 3 Auckland Bioengineering Institute, The University of Auckland, Auckland, New Zealand; 4 Medlink Innovation Limited, Auckland, New Zealand; 5 Department of Pharmacology and Clinical Pharmacology, The University of Auckland, Auckland, New Zealand

**Keywords:** Administration, intranasal, Administration, topical, Drug delivery systems, Nasal airflow, Nasal cavity, Nasal spray, Paranasal sinuses, Sinusitis

## Abstract

**Background:**

Optimising intranasal distribution and retention of topical therapy is essential for effectively managing patients with chronic rhinosinusitis, including those that have had functional endoscopic sinus surgery (FESS). This study presents a new technique for quantifying *in vitro* experiments of fluticasone propionate deposition within the sinuses of a 3D-printed model from a post-FESS patient.

**Methods:**

Circular filter papers were placed on the sinus surfaces of the model. Deposition of fluticasone on the filter paper was quantified using high-performance liquid chromatography (HPLC) assay-based techniques. The deposition patterns of two nasal drug delivery devices, an aqueous nasal spray (*Flixonase*) and metered dose inhaler (*Flixotide*), were compared. The effects of airflow (0 L/min vs. 12 L/min) and administration angle (30° vs. and 45°) were evaluated.

**Results:**

Inhaled airflow made little difference to sinus deposition for either device. A 45° administration angle improved frontal sinus deposition with the nasal spray and both ethmoidal and sphenoidal deposition with the inhaler. The inhaler provided significantly better deposition within the ethmoid sinuses (8.5x) and within the maxillary sinuses (3.9x) compared with the nasal spray under the same conditions.

**Conclusion:**

In the post-FESS model analysed, the inhaler produced better sinus deposition overall compared with the nasal spray. The techniques described can be used and adapted for *in vitro* performance testing of different drug formulations and intranasal devices under different experimental conditions. They can also help validate computational fluid dynamics modelling and *in vivo* studies.

## Introduction

Chronic rhinosinusitis (CRS) represents a spectrum of disorders resulting from complex immunopathological responses that lead to persistent inflammation of the paranasal sinus mucosa. CRS has a prevalence of approximately 10% and has high associated direct and indirect health costs (1). The current recommended medical treatment regimen includes oral antibiotics, sinonasal lavage and systemic and topical corticosteroids.

In patients who fail to respond adequately to medical therapy, functional endoscopic sinus surgery (FESS) is indicated. This procedure opens the obstructed sinus openings (ostia) in order to improve sinus ventilation and restore mucociliary clearance. However, some forms of CRS are driven by incompletely understood self-perpetuating immune-mediated processes. Without long-term topical postoperative medical management, these cases have a significant risk of requiring revision surgery. Topical corticosteroids are the mainstay of postoperative therapy for effective long-term management (2, 3). However, it remains uncertain how efficiently these are delivered to the sinonasal mucosa. Bioavailability is influenced by drug distribution within the sinonasal cavity, absorption across mucosal barriers and the rate of clearance from the nose.

Aqueous nasal sprays are the preferred mode of application of topical nasal medications due to their convenience, simplicity, and dose consistency. Despite this, studies of therapeutic efficacy have yielded inconsistent results, primarily due to the variability of the spray parameters (4, 5) and airway geometries that are unique to each patient (6–9). Even after surgery, local drug delivery to the sinuses remains a challenge due to the persisting complexity of the sinonasal anatomy. The paranasal sinuses are cavities extending outwards from the main nasal passage that are connected by small openings (ostia). The frontal and maxillary sinuses connect at an angle nearly perpendicular to most of the airflow in the nasal passage, presenting further challenges to topical drug delivery (10–12). Nozzle designs, formulations, mode of use and inhalation status are assumed to impact spray volume, distribution, plume shape and spray duration. However, there is conflicting evidence regarding the role of each parameter and understanding of the complex interaction between them remains poor. Optimisation of spray parameters is made difficult by the inability to accurately quantify drug deposition, as the nasal cavity is not easily accessed for drug deposition sampling. *In vivo* studies show a high level of impaction by nasal sprays in the vestibule anterior to the nasal cavity (13–21). Deposition is further restricted by the nasal valve, an elliptical constriction just behind the vestibule, resulting in most particles > 10 µm being trapped anteriorly (22). This region is non-ciliated and so there is a longer residence time. However, the permeability is lower than the mucosa located more posteriorly in the nasal cavity. In contrast to *in vivo* studies, *in vitro* experiments can provide the ability to spatially quantify nasal deposition under well-controlled conditions.

Pressurised metered dose inhalers (MDIs) are generally designed for pulmonary delivery and produce a much smaller particle size than aqueous sprays. After intranasal administration in a healthy, unoperated individual, a large majority of small particles < 10 µm are expected to escape nasopharyngeal capture and travel further into the lower respiratory tract (22). However, the possibility of increased deposition of these smaller particles (< 10 µm) within the sinuses following surgery has not yet been explored (23–26). This has emerged as an area of interest as computational fluid dynamics (CFD) simulations providing highly detailed predictions of flow behaviour and droplet distribution within the sinonasal cavity suggest unique airflow and drug distribution patterns following surgery (23, 25, 26).

This study aimed to design and perform *in vitro* experiments allowing the quantification of topical fluticasone propionate deposition within the sinuses of a single highly detailed three-dimensional (3D) printed model of a post-FESS patient by two commercially available devices: 1) *Flixonase* nasal spray (GlaxoSmithKline, UK) 2) *Flixotide* MDI (GlaxoSmithKline, UK). Filter paper retrieval and high-performance liquid chromatography (HPLC) methods were used. The effects of a limited number of variables (airflow and spray administration angle) were evaluated to enable a better understanding of how surgical strategies affect sinonasal drug distribution and help optimise topical drug delivery to the sinuses.

## Methods

### Physical Sinonasal Model Reconstruction

**Fig. 1:**
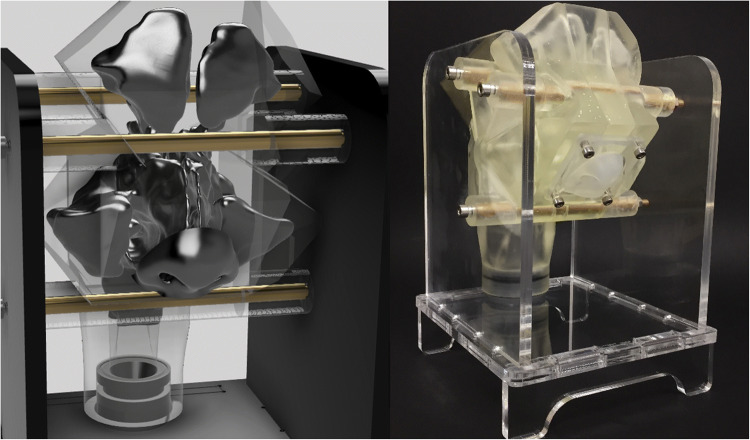
Sinonasal cavity model. Conceptualization (left); printed model (right)

A 3D printed model of a 60-year-old female New Zealand European CRS patient’s post-FESS sinonasal cavity forms the basis of this study (Fig. 1). Written informed consent was obtained from the patient and the study was approved by the New Zealand Health and Disability Ethics Committee.

The 3D surface geometry of the patient’s sinonasal airways was reconstructed from MRI images using segmentation techniques to produce a stereolithography (STL) file. This process is described in detail in our previous publications (23, 26). The STL was repaired using *MeshMixer* software (Autodesk, CA USA) and converted from a tri-mesh to quad-mesh using *Recap Photo* (Autodesk, CA USA). Thickness was added to the surface mesh and sections were created using *Fusion 360* (Autodesk, CA USA). The model was sliced into six sections to be dissembled and provide optimal access to all sinuses. The model sections were printed using *Clear Resin* (Formlabs, MA, USA) on a Form 3 SLA (stereolithography) 3D printer (Formlabs, MA, USA).

**Fig. 2:**
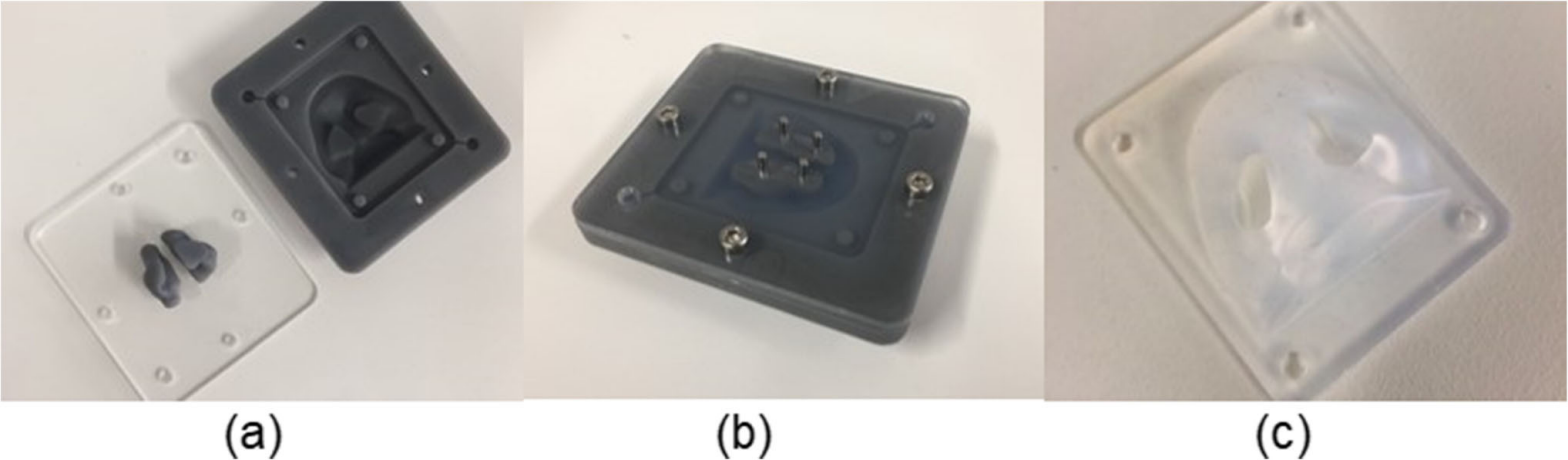
Silicone nose mouldings (a) reverse view of the nasal vestibule region; (b) attachment plate of the mouldings connecting to the main nasal passage; (c) front view of the nostril moulds

**Fig. 3:**
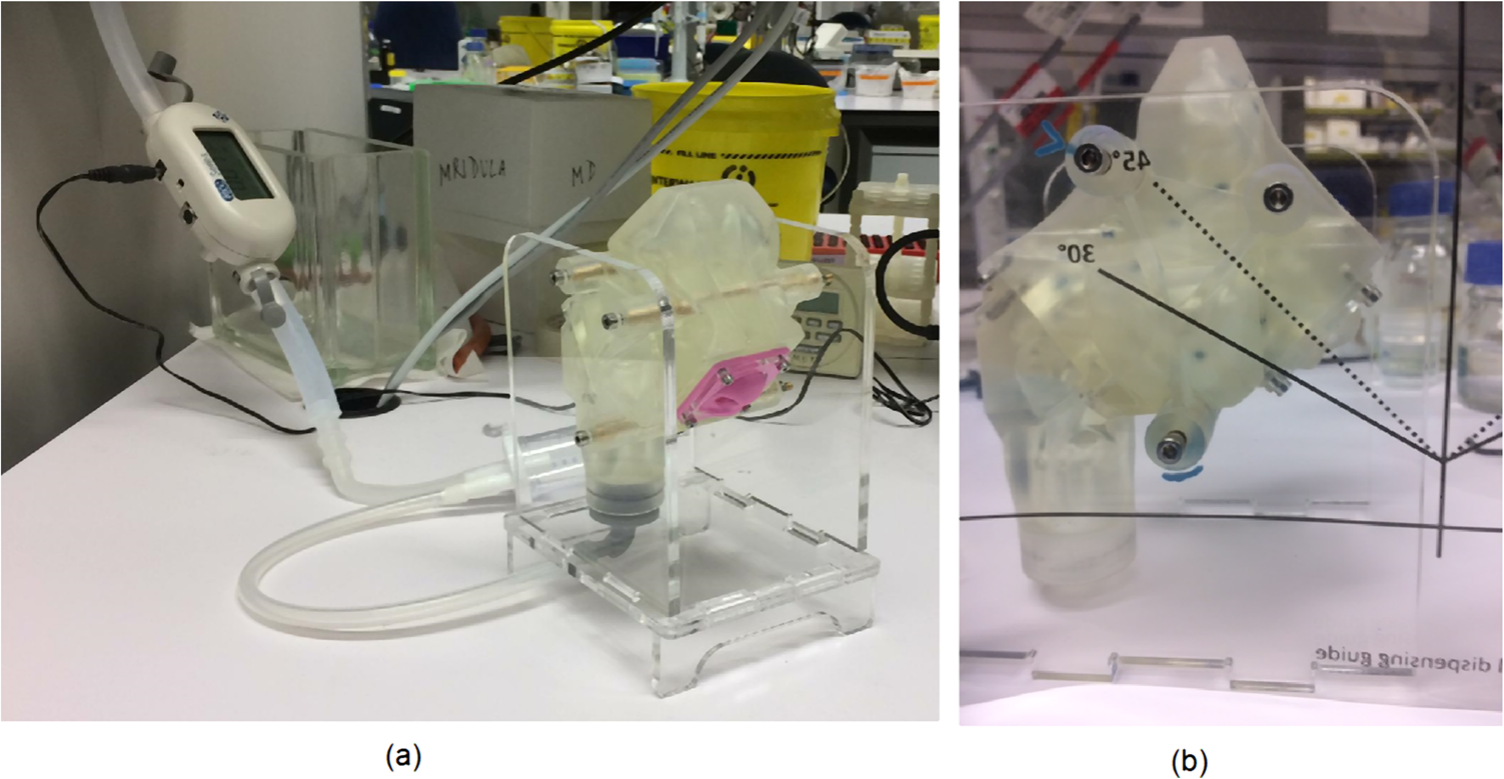
Experiment set up. (**A**) Sinonasal cavity model connected to wall vacuum and flowmeter (**B**) Administration angle dispensing guide

The outer nose was created as an extra component as it needed to be flexible to allow device insertion into each nostril. A model of the patient’s nose was cast using liquid silicone rubber *LSR-4301 Shore 01A* (Elkem, Norway). The mould was constructed from 3D printed using *Grey Pro Resin* (Formlabs, MA, USA) and laser-cut using clear acrylic (Fig. 2). The nose was attached to the sinus model via an acrylic plate clamped over a flange incorporated into the silicone nose. The irregular nasopharynx outlet was lofted to a circular outlet enabling a smooth transition for attaching to a filter compartment (Fig. 1). The filter compartment outlet was connected via a flexible 10 mm internal diameter vacuum line to simulate constant inhalation flow (Fig. 3).

### Quantification Using Limited Sampling Method

**Fig. 4:**
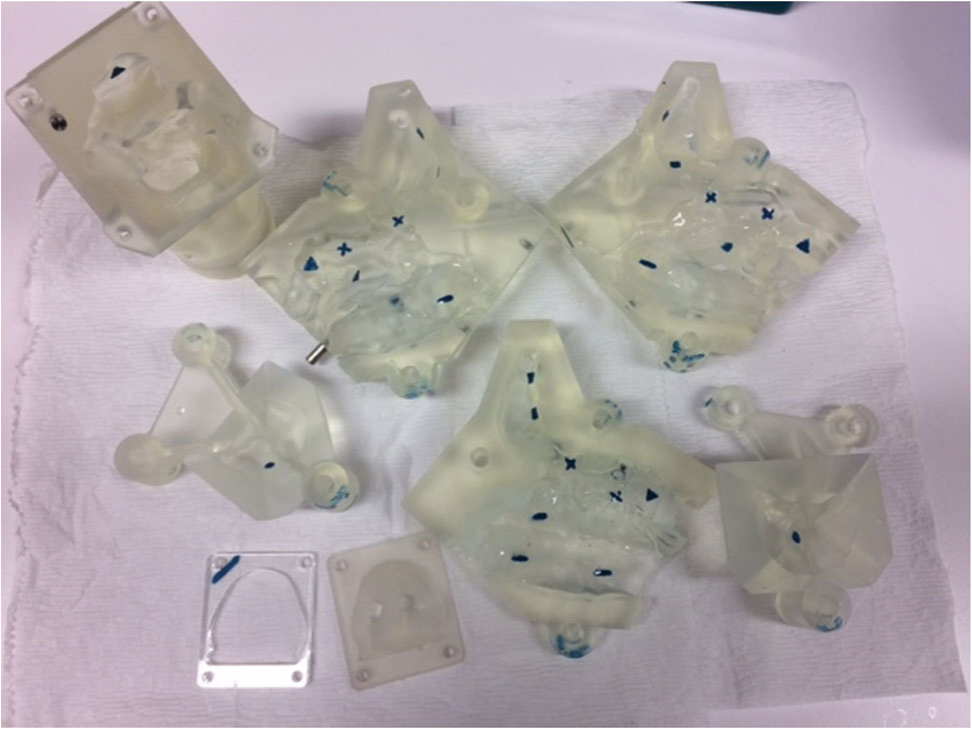
Disassembled sinonasal model with marked locations for filter dot placement in limited sampling technique

The ethmoid, frontal, maxillary and sphenoid sinuses were sampled for drug deposition on both sides of the model using circular filter papers cut from *Whatman®* Grade 1 filter paper (Whatman plc, Maidstone, United Kingdom) placed at marked locations. Four to five locations were selected based on an even distribution in accessible regions and covered approximately 4–15% of each sinus (Table I). Each location on the sinuses was marked with a pen (Fig. 4). Circular dots of 6 mm diameter were dampened with water before being placed in the sinuses, where the filter dots adhered to the sinuses by surface tension. The dampened filter paper acted as a sponge for any drugs that deposited on its surface during the experiments.

**Table I:** Percentage Surface Area Covered by Filter Dots in each Sinus

Sinus	Total surface area (SA) (mm2)	% of SA covered by smaller filter dots in limited sampling technique	% of SA covered by larger filter dots in increased coverage technique
Right ethmoid	1112	10	42
Right frontal	3024	15	53
Right maxillary	3212	4	54
Right sphenoid	2875	4	56
Left ethmoid	977	12	48
Left frontal	2656	4	60
Left maxillary	3241	4	53
Left sphenoid	971	9	49

**Fig. 5:**
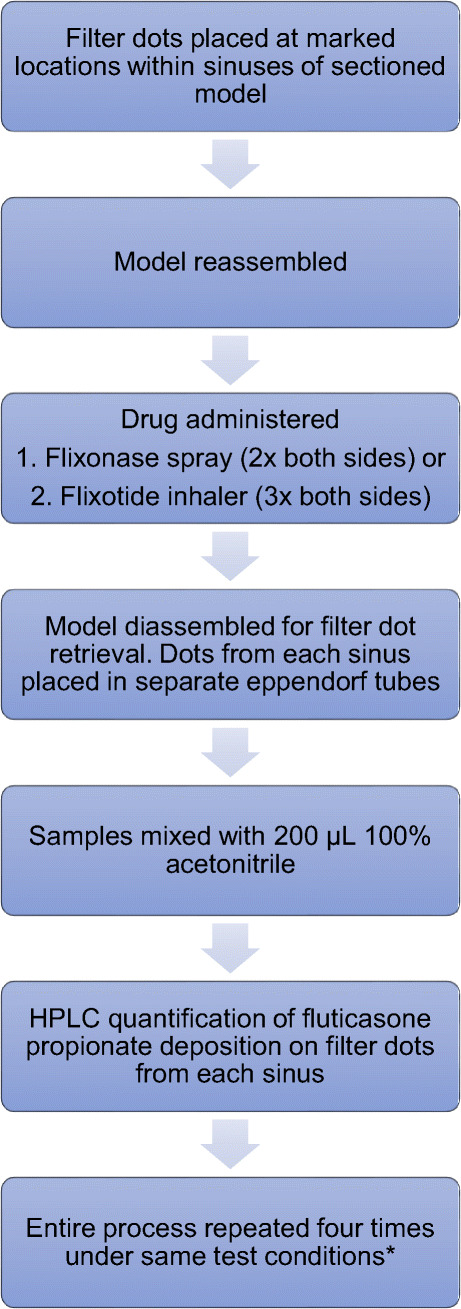
Limited sampling method in which circular filter papers were used to measure drug deposition at selected locations within the sinuses. *Four experimental runs were repeated on each side of the model with a combination of two different flow rates and two different administration angles (0 L/min and 30°, 0L/min and 45°, 12 L/min and 30°, 12 L/min and 45°) giving a total of 32 datapoints

**Fig. 6:**
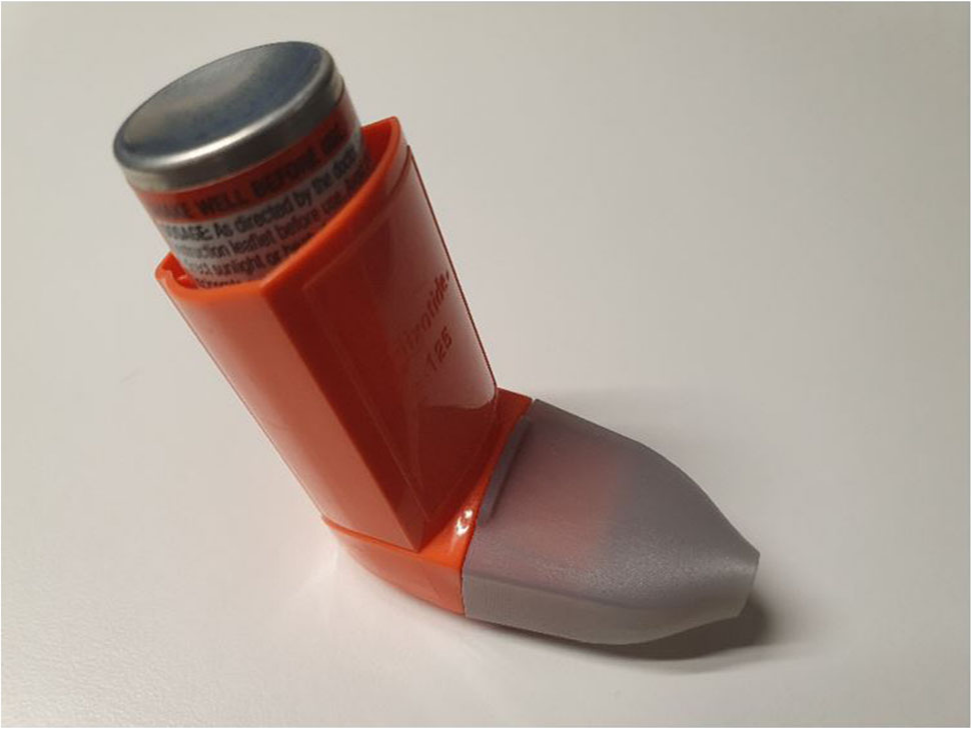
(a) Flixotide MDI with printed nasal adaptor

During the experiments, fluticasone propionate from either a *Flixonase* nasal spray or *Flixotide* metered dose inhaler was administered intranasally in the model under different test conditions (Fig. 5). A nozzle adaptor was designed to allow the MDI to be administered intranasally (Fig. 6). The nozzle adapter was printed using *Tough 1500 Resin* (Formlabs, MA, USA) on a Form 3 SLA (stereolithography) 3D printer (Formlabs, MA, USA). The model was then disassembled for retrieval of the filter dots. Drug elution from the filter dots was performed using 200 µL 100% acetonitrile before quantification using HPLC. The extraction efficiency of a standard solution of fluticasone proprionate from Whatman ® Grade 1 filter paper was measured five times using HPLC. This was consistently greater than 95% and the average extraction efficiency was 97% (SD = 1.7%). In between each experiment, the nasal model pieces were washed twice with 80% ethanol and rinsed twice with laboratory distilled water before being dried using an air blow gun connected to laboratory compressed air.

### HPLC assay

**Fig. 7:**
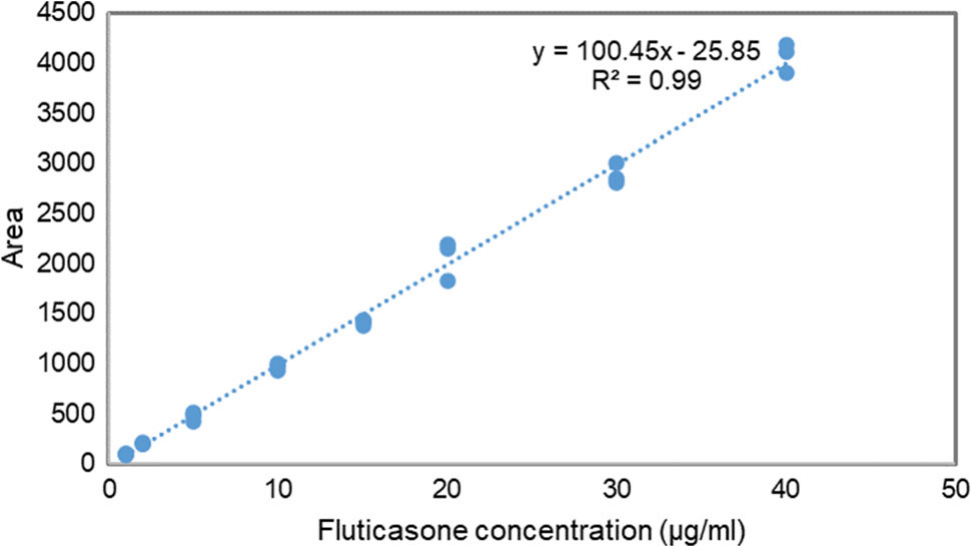
Standard curve obtained to quantify fluticasone propionate

Samples were mixed in a vortex mixer before being centrifuged in a *Heraeus™ Fresco 17* centrifuge (Thermo Fisher scientific, MA, USA) at 12,000 × g for 10 min. The supernatant was analysed using a rapid and sensitive HPLC assay derived from methods described by Couto et al*.* (27) to detect and quantify fluticasone propionate on inhalation particles. A *1260 Infinity II LC System* (Agilent, CA, USA) was coupled with isocratic elution on an Extend-C_18_ column using acetonitrile and water (80:20, v/v) with the flow rate set at 0.5 mL/min. The UV detector was set to 236 nm and the total run time was 5 min. Calibration curves were prepared to assess linearity, precision, and accuracy (Fig. 7). Precision and accuracy were deemed acceptable if measured values were within ± 15% of the actual values (± 20% at the lower limit of quantification, LLOQ). Good linearity (r^2^ = 0.99) was obtained in the range of 0.02 to 0.40 mg/mL for fluticasone propionate.

### Variables Tested

Drug deposition from a *Flixonase* nasal spray and a *Flixotide* MDI was quantified. The total drug recovery from an MDI with a nozzle adaptor (Fig. 6) was measured five times using the HPLC assay. The average total drug recovery from the MDI was 63% (SD = 2%) as some drug particles were retained on the inside of the adaptor. In each experimental run, the *Flixotide* MDI was administered three times successively (approx. 225 µg), while the *Flixonase* nasal spray was administered twice successively (approx. 100 µg). The spray was only administered twice to minimise dribbling of the drug within the model anteriorly.

Four experimental runs were performed on each side of the model with a combination of two different flow rates and two different administration angles (0 L/min and 30°, 0L/min and 45°, 12 L/min and 30°, 12 L/min and 45°) giving a total of 32 data points (Fig. 5). The administration angle was maintained using a printed dispensing guide (Fig. 3b). A constant flow rate of 12 L/min was applied by attaching the filter compartment to wall suction. The airflow rate and pressure gradient remained stable as measured on a TSI 4000 flowmeter (TSI, MIN, USA). An inhalational flow rate of 12 L/min was chosen to reflect the maximum flow rate during the initiation phase of inhalation. Although peak inhalational flow rate is approximately 18 L/min during restful breathing (28), the initial particle velocity of nasal sprays and MDIs is so high (29) that particle deposition most likely occurs in the initial phase of inhalation (< 10 L/min). This assumption also holds true for sniffing (28).

The average % drug deposition was calculated:

% drug deposition = quantity of drug deposited on filter dots / total quantity of drug administered × 100.

The paired student’s *t*-test was used to analyse differences between variables.

### Quantification Using Increased Coverage Sampling Method

**Fig. 8:**
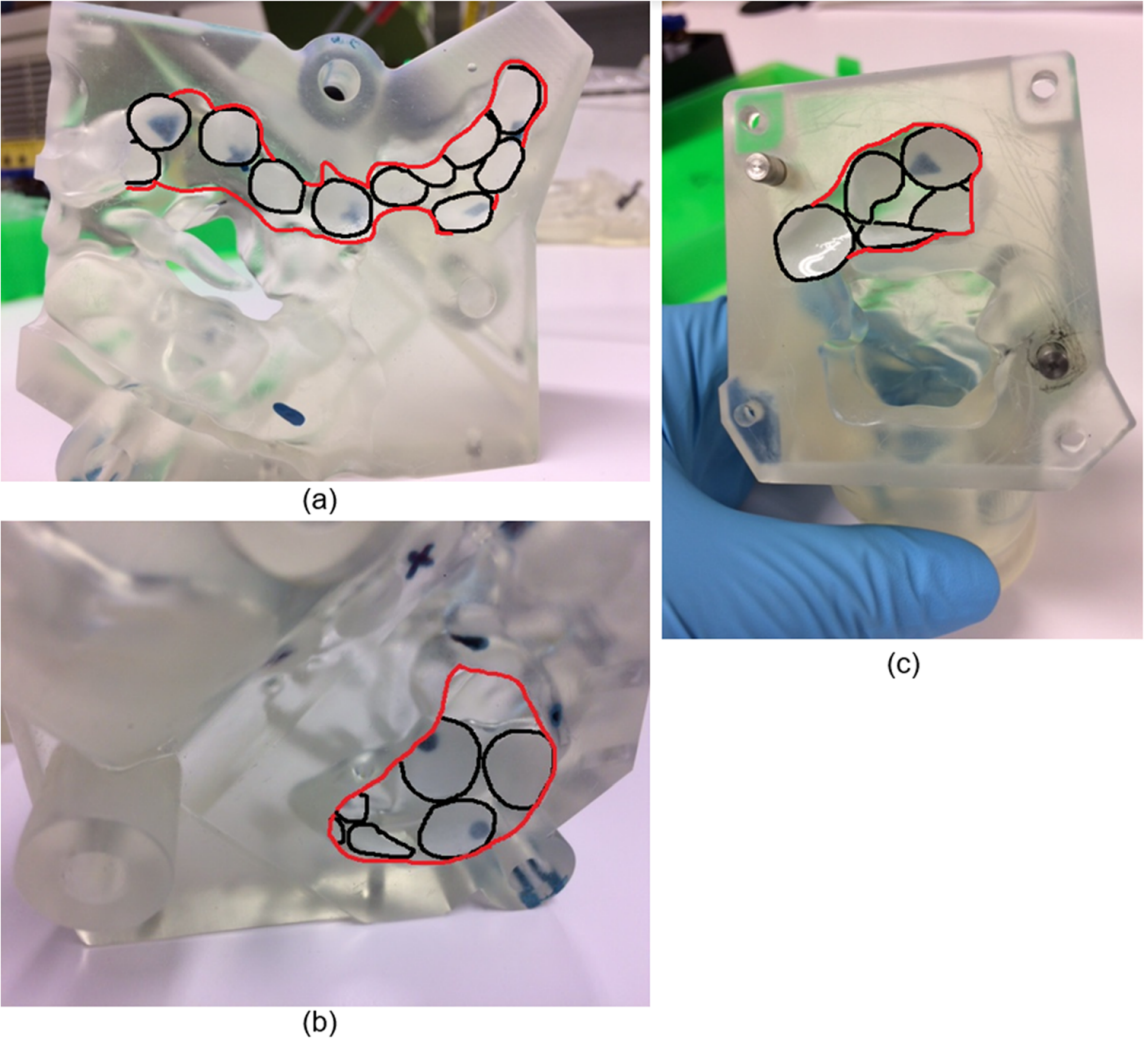
Filter dot placement for drug quantification using increased coverage sampling method. (A) Sagittal view showing medial surfaces of right frontal, ethmoid and sphenoid sinuses (B) Medial surface of right maxillary sinus (C) Posterior right sphenoid sinus. Red = sinus region outline; black = filter dot borders

An increased coverage method was employed to confirm whether one device had superior sinus deposition while applying the optimal variables (device administration angle and flow rate) determined from the limited sampling technique for each device. Instead of using 6 mm filter dots in the marked locations, larger circular dots of 10–12 mm diameter were placed closer together in each sinus region to uniformly and maximally cover the surfaces (Fig. 8). For each device, three experimental runs were repeated on each side of the model.

Between 42- and 60% of each sinus region was covered with larger dots using the increased coverage method (Table I). Data were collected for each sinus region from six tests that consisted of three tests repeated on each side of the model. This was used to calculate the average % drug deposited on the filter media. The mean estimated % total drug deposition for each sinus was then calculated based on the proportion of total surface area (SA) covered by the filter media in each region.

Mean estimate % total drug deposition = average % drug deposited /% SA of region covered × 100.

## Results

### Optimal Flow Rate and Angle For Sinus Deposition Using Limited Sampling

Table II shows the optimal flow rate and angle for the nasal spray and MDI for sinus deposition collected by the filter paper. A total of 16 data points were collected for each site (ethmoid, frontal, maxillary, and sphenoid sinuses) with no inhalation (0L/min) and at a flow rate of 12L/min, as well as 16 data points for each angle of administration (30°and 45°).

An inhalation flow of 12 L/min did not improve sinus deposition for either the nasal spray or the MDI compared to no flow (0 L/min). In contrast, better deposition occurred in the sphenoid sinuses with an MDI (*p* = 0.03) with no flow (0 L/min). A 45° administration angle improved frontal sinus deposition with a nasal spray (*p* = 0.001) and both ethmoidal and sphenoidal deposition with an MDI (*p* < 0.005) compared to a 30° administration angle.

### Optimal device for sinus deposition using limited sampling.

Table III shows a comparison between the nasal spray and MDI for sinus deposition based on the 32 data points for each site (ethmoid, frontal, maxillary and sphenoid sinuses) for the *Flixonase* spray and *Flixotide* inhaler under the different test conditions. The nasal spray had better improved deposition within the frontal sinuses (*p* = 0.03) whereas the MDI had better deposition within the ethmoid sinuses (*p* = 0.001).

### Drug Deposition Measured by Increased Coverage Sampling

A further comparison between the devices was made using an increased coverage sampling method to confirm whether one device had superior sinus deposition. This method was employed while applying the optimal variables (device administration angle 45° and flow rate 0 L/min), determined from the limited sampling technique for the devices. The average % of drug deposition on the larger filter paper dots was calculated from six runs performed for each region (including three runs repeated on each side of the model). The estimated % total drug deposition was calculated based on the proportion of surface area covered by filter dots in each region. The estimated % total drug deposition for the nasal spray and MDI in each sinus is shown in Table IV. Using this method, the MDI was found to provide 8.5 × better deposition within the ethmoid sinuses (*p* = 0.02) and 3.9 × better deposition within the maxillary sinuses (p = 0.01) compared with the nasal spray. The MDI tended to provide better deposition within the frontal and sphenoid sinuses, but this difference was not statistically significant.

## Comparison Between Sampling Methods

A comparison of the estimated % total drug deposition between the limited sampling method and increased coverage method using the investigated variables (device administration angle 45° and flow rate 0L/min) is given in Table V.

The estimated % total drug deposition for the nasal spray for each region was higher using the limited sampling method compared with the increased coverage method, suggesting that the marked locations for limited sampling received a disproportionally higher quantity of drug from the nasal spray than the remaining unsampled locations. This resulted in likely overestimation of total drug deposition caused by non-uniform particle deposition. The estimated % total drug deposition for the two sampling methods was more similar for the MDI suggesting more uniform particle deposition.

## Discussion

Benchtop techniques for characterising spray properties in an unrestricted laboratory test environment are generally poor at predicting deposition in a nasal cast or human nose (30, 31). *In vitro* nasal cast deposition measurements potentially provide data that are more closely representative of spray behaviour in the nasal cavity. There is not currently a quantitative *in vivo* distribution method that provides an accurate three-dimensional representation of regional drug distribution without using radioisotopes or high-energy radiation (31, 32).

The initial experiments described in this study sampled a smaller area to quantify drug deposition in locations selected to be evenly distributed in each sinus region. This method aimed to determine the optimal flow rate (0 L/min vs. 12 L/min) and angle of administration (30° vs. 45°) for sinus deposition of *Flixonase* nasal spray and *Flixotide* MDI. In the following experiments where sampling was obtained from a much wider surface area, the aim was to determine whether the nasal spray or the MDI achieved better sinus deposition under the established optimal conditions (0 L/min flow rate and 45° angle of administration). Results showed that less than 1% of the nasal spray deposited within each sinus region even under optimised conditions. Poor sinus deposition and efficacy with nasal sprays can be attributed to the delivery of large droplets travelling at high velocities (32, 33) that deposit in the anterior nasal cavity (34–37), thereby failing to navigate through the narrowed passages to reach the affected sinus mucosa. In addition, many patients who use nasal sprays harbour concerns of bleeding, diminished effectiveness due to the dripping of the formulation out of the nose or the unpleasant taste attributed to dripping down the throat.

The MDI produced better results overall under the same conditions, with a greater % of total drug deposition within the sinuses (up to 7.3% in the ethmoid sinuses, 1.7% in the maxillary sinuses and 2.8% in the sphenoid sinuses). Findings from a CFD study by this group showed that deposition within the sinuses is generally more effective with low-inertia particles outside of the range produced by many standard nasal sprays or nebulisers (26). This effect becomes more pronounced with increasingly extensive surgery, as the sinus and nasal cavity become more interconnected and functionally interdependent and sinus aeration is enhanced after FESS (23, 26). However, this study showed that inhalation from MDIs appears to have little influence on sinus deposition. Even though inspiratory flow likely transports drug particles deeper into the sinonasal cavities, this effect is probably negated by more particles escaping into the lower respiratory tract upon inhalation (22). The observed improvement in sinus deposition with MDIs is more likely attributed to their narrower plume angle (< 20°) (38, 39) and wider and more uniform dispersion of small particles (further facilitated by FESS). A narrower plume allows better drug penetration through the narrow nasal valve (30, 35, 40). Despite this, the high initial velocities of particles produced by MDIs still present significant limitations to sinonasal deposition (41–44). Exhalation delivery systems appear to provide enhanced intranasal deposition as a patient’s exhaled breath becomes the main transport mechanism for drug particle delivery (45).

Finally, frontal sinuses remain challenging for any device to penetrate due to the particularly narrow frontal sinus drainage pathway. Access is improved in patients who have had more extensive frontal sinus surgery (Modified Lothrop Endoscopic Procedure) (26, 45).

### Limitations

The generalisability of this study is limited since only a single post-FESS model was studied. Variations in both nasal anatomy and extent of surgical intervention between patients can influence sinonasal deposition. The purposes of this study were firstly to design and test an inexpensive method to quantify fluticasone propionate deposition patterns in an anatomically correct postoperative sinonasal cavity model and secondly to demonstrate the utility of this approach by comparing the deposition patterns of two different commercially available nasal drug delivery devices and assessing the effect of a limited number of variables. Variables such as the depth of device insertion and head position were not evaluated. Although the constant inspiratory flow applied in this study did not simulate the physiological breathing cycle, inhalation flow appeared to have little effect on drug deposition patterns. The limited sampling technique was a more time-efficient method of determining the optimal parameters for each device but was inferior to the increased coverage sampling method in comparing total drug deposition between the devices.

Nasal casts are simplified representations of human anatomy, lacking biological surface properties and mucociliary clearance. In this study, the difficulty of drug penetration through the nasal valve may be underestimated since a silicone nose and rigid cast does not re-create the dynamic narrowing of the tissue of the anterior part of the nasal valve region during the nasal cycle and with breathing. Furthermore, *in vitro* tests are limited in their ability to predict pharmacokinetic properties. However, the very significant advantage of using a nasal model is the ability to tightly control experimental conditions. As such, The U.S. Food and Drug Administration recommends *in vitro* techniques for the optimisation of device design parameters and formulations (46).

Spray-visualization using a *Sar-gel* (Arkema, Colombes, France) colour-based method is a semi-quantitative and less expensive method that has been used successfully (45, 47). However, this methodology relies on a certain quantity of water-based formulation deposition for colour change, and quantification is based on lateral 2D images within defined boundaries. *Sar-gel* does not change colour sufficiently on exposure to fluticasone to be used in our model. The novel methodology detailed in this study was developed to quantify fluticasone deposition from two commercially available devices, *Flixonase* and *Flixotide*. As with the *Sar-gel* method, this technique required no adulteration of the devices or formulations. The sinonasal cavity model was designed to suit the methodology, and we do not suggest that the model we used is superior to other nasal casts or representative of all nasal geometries.

## Conclusion

An *in vitro* method has been developed to quantify fluticasone propionate drug deposition patterns in an anatomically correct postoperative sinonasal cavity model. This has been used to compare the deposition patterns of two different commercially available nasal drug delivery devices: an aqueous nasal spray and metered dose inhaler. In the patient model analysed, the metered dose inhaler provided greater sinus deposition. The techniques described can be used and adapted for *in vitro* performance testing, of different drug formulations and devices under different experimental conditions. This can help validate other forms of *in vitro* testing, CFD and *in vivo* validation studies.

**Table II:** Optimal Flow Rate and Angle for Nasal Spray and MDI for Sinus Deposition at Marked Sites

Device	Site	Flow rate^§^ (0 vs. 12 L/min)	Diff.*	P value	Spray angle^§§^ (30° vs. 45°)	Diff.*	P value
Spray	Ethmoid	*NS*	*n/a*		*NS*	*n/a*	
	Frontal	*NS*	*n/a*		45°	1.5x	**0.001**
	Maxillary	*NS*	*n/a*		*NS*	*n/a*	
	Sphenoid	*NS*	*n/a*		*NS*	*n/a*	
MDI	Ethmoid	*NS*	*n/a*		45°	2.7x	**0.004**
	Frontal	*NS*	*n/a*		*NS*	*n/a*	
	Maxillary	*NS*	*n/a*		*NS*	*n/a*	
	Sphenoid	0 L/min	1.6x	**0.003**	45°	1.3x	**0.002**

NS = no significant difference was found between two parameters tested.

^§^16 datapoints analysed for each site. These data were collected from 4 runs repeated on each side of the model at two different angles (30° and 45°).

^§§^16 datapoints analysed for each site. These data were collected from 4 runs repeated on each side of the model at two flow rates (0 and 12 L/min).

*Diff = difference in % drug deposition of device with optimal variable compared with less optimal variable.

**Table III:** Optimal Device for Sinus Deposition at Marked Sites

	Optimal device (diff.)* Flow rate 0L/min, both angles^§^	P value	Optimal device (diff.)* Flow rate 12L/min, both angles^§^	P value	Optimal device (diff.)* Both flow rates and spray angles^§§^	P value
Site	*NS*		*NS*		*NS*	
Ethmoid	*NS*		*NS*		MDI (1.5x)	**0.03**
Frontal	Spray (1.6x)	**0.007**	Spray (1.3x)	**0.007**	Spray (1.4x)	**0.0001**
Maxillary	*NS*		*NS*		*NS*	
Sphenoid	*NS*		*NS*		*NS*	

NS = no significant difference was found between spray and MDI.

^§^16 datapoints analysed for each site. These data were collected from 4 runs repeated on each side of the model at two different angles (30° and 45°).

^§§^32 datapoints analysed for each site. These data were collected from 4 runs repeated on each side of the model at two different angles (30° and 45°) and two different flow rates (0 and 12 L/min).

*Diff = difference in % drug deposition of optimal device compared to % drug deposition of less optimal device.

**Table IV:** Estimated % Total Drug Deposition for Nasal Spray and MDI in each Sinus using Increased Coverage Sampling Method

	Spray			MDI			MDI vs. spray	
	Average % drug deposited^§^	SD	Mean estimate % total drug deposition*	Average % drug deposited^§^	SD	Mean estimate % total drug deposition*	Diff.**	P value
Ethmoid	0.39	0.02	0.86	3.30	2.9	7.3	8.5x	0.02
Frontal	0.27	0.03	0.49	0.42	0.3	0.7	1.5x	0.40
Maxillary	0.26	0.03	0.49	0.92	0.4	1.7	3.5x	0.01
Sphenoid	0.38	0.02	0.71	1.50	2.7	2.8	3.9x	0.20

R = right; L = left.

^§^Average result of 6 runs performed for each site including 3 runs repeated on each side of the model.

* Mean estimate % total drug deposition was calculated based on the proportion of surface area (SA) covered by filter medium in each region; mean estimate % total drug deposition = (average % drug deposited /% SA of region covered by filter dots) × 100.

**Diff = difference in mean estimate % total drug deposition of MDI compared to that of nasal spray.

**Table V:** Comparison of Mean Estimate % Total Drug Deposition using Limited Sampling versus Increased Coverage Sampling Methods

	Mean estimate % total drug deposition for spray*		Mean estimate % total drug deposition for MDI	
Site^§^	Limited sampling	Increased coverage sampling	Limited sampling	Increased coverage sampling
Ethmoid	3.1	0.9	6.9	7.3
Frontal	1.5	0.5	1.0	0.7
Maxillary	5.2	0.5	4.7	1.7
Sphenoid	3.0	0.7	3.8	2.8

* Mean estimate % total drug deposition was calculated based on the proportion of surface area (SA) covered by filter medium in each region; mean estimate % total drug deposition = (% average drug deposited /% SA of region covered) × 100.
